# A Stem Cell Surge During Thyroid Regeneration

**DOI:** 10.3389/fendo.2020.606269

**Published:** 2021-01-21

**Authors:** Risheng Ma, Syed A. Morshed, Rauf Latif, Terry F. Davies

**Affiliations:** Thyroid Research Unit, Department of Medicine, The Icahn School of Medicine at Mount Sinai and the James J. Peters VA Medical Center, New York, NY, United States

**Keywords:** ROSA26iDTR mice, TPOCreER2 mice, stem cells, thyroid, regeneration

## Abstract

**Background:**

Many tissues, including the thyroid, contain resident (adult) stem cells that are responsible for regeneration and repair after injury. The mechanisms of thyroid regeneration and the role of thyroid stem cells and thyroid progenitor cells in this process are not well understood. We have now used a new mouse thyroid injury model to gain insight into this phenomenon.

**Methods:**

Tamoxifen induced TPO-Cre mice (TPOCreER2) were crossed with inducible Diphtheria Toxin Receptor homozygous mice (ROSA26iDTR) to give rise to TPOCreER2/iDTR mice, allowing for the Cre-mediated expression of the DTR and rendering TPO expressing thyroid cells highly sensitive to diphtheria toxin (DT). This model of TPOCreER2/iDTR mice allowed us to study the repair/regeneration of thyroid follicles after diphtheria toxin induced thyroid damage by measuring serum thyroid hormones and cell fate.

**Results:**

In TPOCreER2/iDTR double transgenic mice we observed severe thyroid damage as early as 2 weeks after initiating intraperitoneal DT injections. There was marked thyroid tissue apoptosis and a ~50% drop in serum T4 levels (from 5.86 to 2.43 ug/dl) and a corresponding increase in serum TSH (from 0.18 to 8.39 ng/dl). In addition, there was a ~50% decrease in transcription of thyroid specific genes (thyroglobulin, TSH receptor, and sodium-iodide symporter). After suspending the DT administration, the thyroid rapidly recovered over a 4-week period during which we observed a transient surge in stem cell marker expression (including Oct4, Nanog, Sox2, and Rex1). In addition, cells immunostaining with stem cell markers Oct4 and Ssea-1 were found in clusters around new thyroid follicles in TPOCreER2/iDTR double transgenic mice. Furthermore, the presence of clusters of thyroid progenitor cells was also identified by Pax8 staining of thyroglobulin negative cells. This recovery of the injured gland was followed by a rapid and sequential restoration of thyroid function.

**Conclusion:**

These data demonstrate that a new model of thyroid cell damage induced by DT can be used to study the mobilization of resident adult stem cells. Furthermore, the model clearly demonstrates the involvement of both stem and progenitor cells in the *in vivo* regeneration of the thyroid after severe destruction.

## Highlights

The capacity of the adult thyroid gland to regenerate in response to injury represents an important homeostatic process. In this study, we have used a new mouse thyroid injury model to demonstrate that stem cells are mostly responsible for regeneration and repair after an insult. The model clearly demonstrates the involvement of both stem and thyroid progenitor cells in the *in vivo* regeneration of the thyroid gland after severe destruction and likely models the human thyroid response to injury.

## Introduction

The thyroid gland is a relatively dormant organ with a low cell turnover rate and with follicular cells which only divide ~ﬁve times in adult life (1, 2). However, the thyroid gland does have the capacity to grow through cell hypertrophy and/or proliferation in response to hypothyroidism caused by various stimuli including TSH, xenobiotics, or pathophysiological conditions (i.e. iodide deficiency, autoimmune disease, partial thyroidectomy, etc.) in order to restore normal thyroid hormone levels (3, 4). The mechanisms of this restorative function of the thyroid has been exploited by using partial thyroidectomy, experimental autoimmune thyroiditis (EAT), or transgenic approaches in mice as a tool to study thyroid regeneration (3, 5–8). However, the mechanisms involved remain to be fully characterized and the relative roles of follicular cell hyperplasia and stem cell activation remain unclear (7, 8). The control of regeneration of a thyroid gland is complex. Various stimuli including xenobiotics, growth factors and physiological alterations that perturb the pituitary-thyroid axis and increase the levels of TSH may stimulate immature micro-follicles found in thyroid glands to mature into functional thyroid cells and rescue the hormone levels (6). Such immature thyroid cells may be resident stem cells or resident progenitor thyroid cells that are able to repair/regenerate a damaged thyroid gland. However, without the appropriate markers being identified such reports remain largely speculative (1, 5, 6, 9).

Thyroid stem cells are now recognizable by their development into progenitor cells which simultaneously express two major transcription factors, Pax-8 and NKX2-1 (TTF1) which are only expressed together in thyroid cells (10–12) but which have not yet differentiated to express thyroid specific genes. While the presence of stem cells in the thyroid gland has now been recognized (9, 13–15) the role of such cells in thyroid hypertrophy and hyperplasia remains largely uncharacterized. Animal models should be ideal to explore the mechanisms of thyroid repair but previous models have failed to fulfill this promise. For example, the thyroid tissue in murine experimental autoimmune thyroiditis (EAT) exhibited stem cell markers during the recovery phase but this model is complicated by the aggressive immune response to immunization (8). Mouse models of partial thyroidectomy have been used to show hyperplasia and the presence of stem cells in the remaining thyroid lobe but by definition the remaining thyroid was not regenerative but undergoing hypertrophy (7). A model using NKX2-1 KO mice has also been employed to implicate stem cells but in this hypothyroid model none of the cells were able to differentiate into functional thyroid cells because of the absence of NKX2-1 (16).

In order to study the role of resident thyroid stem cells under native regenerative conditions we have developed a new mouse model of controlled thyroid cell damage to see if this approach activates stem cells and lineage-committed progenitor cells leading to restoration of thyroid function. The Cre-inducible Diphtheria Toxin Receptor (iDTR) transgenic mouse, in which Cre-mediated excision of a STOP cassette renders cells sensitive to Diphtheria Toxin (DT) (17), appeared to be just such a potential model. We have used a thyroid peroxidase (TPO) CreER2 transgenic mouse which expresses Cre-ERT2 inducible by tamoxifen (TM) under the direction of the human TPO promoter, and which, when treated with tamoxifen, deletes the floxed stop sequence only in the thyroid cells expressing TPO. Thus, we developed the TPOCreER2/iDTR model by crossing the TPOCreER2 mice to ROSA26iDTR and induced damage of the TPO-expressing thyroid gland cells by intraperitoneal injection of both TM and DT. In this system, any non-TPO expressing cells, such as stem cells and progenitor cells, are unaffected since TPO is a late gene and seen only in mature thyroid follicular cells. Using this model we have identified a sequential stem cell surge after thyroid damage indicating that resident stem cells are intimately involved in the restoration of the thyroid gland.

## Materials and Methods

### Mice and Maintenance

**TPOCreER2** transgenic mice (Jackson Laboratory, Bar Harbor, ME, USA, JAX stock #026512) (18) express Cre-ERT2 under the direction of the human thyroid peroxidase (TPO) promoter making them ideal for applications requiring **tamoxifen-induced** deletion of floxed stop sequences in the thyroid. **ROSA26iDTR** mice (Jackson Laboratory, Bar Harbor, ME, USA, JAX stock #007900) (19), have Cre-inducible expression of DTR (iDTR knock-in) rendering them susceptible to ablation following Diphtheria toxin administration. We crossed the ROSA26iDTR mice to the TPOCreER2 to generate **TPOCreER2/iDTR** mice which could serve as a model for depletion of TPO-expressing thyroid gland cells. First generation mice were genotyped using DNA from tail biopsies according to the Jackson Laboratory protocols (**Supplementary Figure 1**). Expression of DTR in the thyroid glands was confirmed by qPCR (**Supplementary Figure 2**). The TSHR-KO control mice were maintained as previously described (20). All mice were kept in a specific pathogen-free barrier at the central animal facility of the Icahn School of Medicine at Mount Sinai. The experiments were performed in accordance with guidelines of the Institutional Animal Care and Use Committees of the Icahn School of Medicine at Mount Sinai.

### Tamoxifen (TM) and Diphtheria Toxin (DT) Preparation and Administration

TM (Sigma-Aldrich, St. Louis, MO, USA, T5648) was dissolved in filter-sterilized corn oil (Sigma-Aldrich, St. Louis, MO, USA, C8267) placed on a nutator to make solutions of 10 mg/ml, which were subsequently protected from light. TM solutions were freshly prepared the day prior to each injection. The DT (Sigma-Aldrich, St. Louis, MO, USA), a lyophilized 1 mg, was resuspended with saline at 1 mg/ml (and aliquoted at 10 ul per tube and frozen at −80°C).

### Injection Protocol

**Figure 1:**
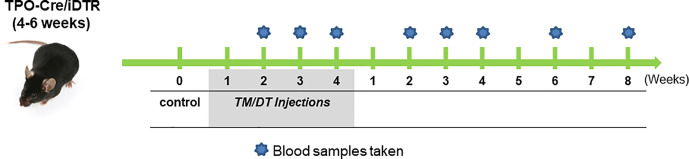
The experimental protocol. TPO-Cre/iDTR mice aged 4–6 weeks received intraperitoneal injection of diphtheria toxin (0.1 ug/gm body weight) and tamoxifen (1 mg/100 ul/mouse) every other day in weeks 1–4 as illustrated. Blood samples were collected at the indicated time points during the injection period and after stopping the injections. Samples were then subjected to TSH and total T4 in serum assays, their thyroids were collected from randomly chosen mice for histology and also for total thyroid RNA preparation for transcriptional analysis.

For thyroid follicular cell ablation, TPOCreER2/iDTR mice, 4–6 weeks of age, were injected intraperitoneally every other day with 1 mg TM (100 ul/mouse) and 100 ng DT/gram body weight in groups of five mice. TPOCreER2/iDTR mice were injected with same volume of vehicle as control. Injection sites were sealed with wet bond tissue adhesive (3M) to prevent oil leakage. Mice were treated with the above regime for 4 weeks and then the injections were discontinued and the mice followed for a further 8 weeks (see **Figure 1** for detailed protocol). Following administration, mice were housed individually to prevent cross-contamination. The results expressed were from two independent experiments.

### Histology Analyses

Mice were sacrificed and thyroid glands were harvested using a steromicroscope at different time points pre and post TM/DT administration and ﬁxed in 4% paraformaldehyde in PBS and embedded in paraffin. After being processed, the sections were stained with hematoxylin-eosin (H&E) to examine the gross histological changes. To evaluate thyroid cell apoptosis, TUNEL staining was performed on 6 μm paraffin sections of thyroid tissue using an “In Situ Cell Death Kit” (Roche Applied Science, Mannheim, Germany). The antigen retrieval (2.5% proteinase K at 37°C) and staining process followed the manufacturer’s instructions and DAPI was used for counter-staining. TUNEL-positive cells were imaged under a fluorescent microscope and the FITC-labeled positive cells were quantitated by measuring fluorescence intensity (FI) using Adobe Photoshop. FI was calculated using corrected total cell fluorescence (CCTF) as described here CCTF = Integrated intensity − (Area of selected cell × Mean fluorescence of background readings). Three positive images were counted in each group and mean and SD was plotted as indicated in the graphs. To perform immunostaining, paraffin embedded sections of thyroid gland were deparaffinized and rehydrated, antigen retrieval was performed using a microwave oven in citrate-based solution (Vector Laboratories Inc, Cambridge, MA, USA). After incubating in blocking buffer (1% horse serum in PBS) for 1 h at room temperature, the sections were incubated with appropriate primary antibodies (for details see **Table 1**) in 1% horse serum, 0.3% Triton X-100, and 1% BSA in PBS overnight at 4°C. Sections were rinsed three times with PBS and incubated with the appropriate secondary antibody (**Table 1**) and then washed twice with PBS, and mounted using hard set mounting media containing DAPI (Vector Laboratories, Burlingame, CA, USA). Slides from each group were incubated with the appropriate secondary antibody used as a negative control. Fluorescence micrographs were acquired using a fluorescent confocal microscope and images were processed and assembled in Photoshop (Adobe, San Jose, CA, USA).

**Table 1:** Antibodies used in the described Methods.

	Name	Host	Source	Catalog #	~Dilution	
					Immunofluorescence	Flow Cytometry
**Primary Antibodies**	Polyclonal to OCT4 antibody	Rabbit	Cell Signaling Technology, Danvers, MA	2750	1:200;	1:200
	Monoclonal to SOX2 antibody	Rabbit	Cell Signaling Technology, Danvers, MA	3579	1:400	1:300
	Polyclonal to NANOG antibody	Rabbit	Cell Signaling Technology, Danvers, MA	3580	1:800	1:400
	Monoclonal to SSEA-1 antibody	Mouse	Thermo Fisher Scientific, Waltham, MA	41-1200	1:500	1:500
	Monoclonal to NKX2-1 antibody	Rabbit	Abcam, Cambridge, UK	ab76013	1:400	1:500
	Monoclonal to PAX8 antibody	Mouse	Thermo Fisher Scientific, Waltham, MA	MA1-117	1:100	1:100
	Monoclonal to TG antibody	Rabbit	Abcam, Cambridge, UK	ab156008	1:200	–
**Polyclonal Secondary Antibodies**	Anti-rabbit IgG (H+L), F(ab’)2 Fragment (Alexa Fluor^®^ 488 Conjugate)	Goat	Cell Signaling Technology, Danvers, MA	4412	1:1,000	1:1,000
	Anti-mouse IgG (H+L), F(ab’)2 Fragment (Alexa Fluor^®^ 488 Conjugate)	Goat	Cell Signaling Technology, Danvers, MA	4408	1:1,000	1:1,000
	Anti-rabbit IgG (H+L), F(ab’)2 Fragment (Alexa Fluor^®^ 555 Conjugate)	Goat	Cell Signaling Technology, Danvers, MA	4413	1:1,000	1:1,000
	Anti-mouse IgG (H+L), F(ab’)2 Fragment (Alexa Fluor^®^ 555 Conjugate)	Goat	Cell Signaling Technology, Danvers, MA	4409	1:1,000	1:1,000
	Anti-rabbit IgG (H+L), F(ab’)2 Fragment (Alexa Fluor^®^ 647 Conjugate)	Goat	Cell Signaling Technology, Danvers, MA	4414	1:1,000	1:1,000

### Thyroid Function Testing

Serum samples were diluted 1:6 in assay buffer and measured for T4 and TSH levels using the Millipore MAP thyroid magnetic bead method (Cat # RTHYMAG-30 K) as per the manufacturer’s protocol. The cut-off for each assay was specified by ±2 standard deviations from the average of the control empty vector immunized samples and was T4 60.1 ± 0.61 ng/ml and TSH 0.25 ± 0.10 ng/ml.

### Gene Expression Analysis

Mice were sacrificed and dissected at different time points as indicated in **Figure 1**. After isolation and removal *en bloc*, total RNA of the thyroid glands was extracted using RNeasy kits (Qiagen Ltd, Manchester, UK) and treated with ribonuclease-free deoxyribonuclease (Qiagen Ltd, Manchester, UK). Five micrograms of total RNA were reverse transcribed into cDNA using the SuperScript III system (Invitrogen, San Diego, CA, USA). Real-time quantitative RT-PCR (qRT-PCR) (see the primers listed in **Supplementary Table 1**) was carried out using a SYBR green qPCR master mix (Applied Biosystems, Foster City, CA, USA) employing the StepOnePlus system (Applied Biosystems, Foster City, CA, USA). Relative expression levels of each gene in real-time were analyzed using the 2^-ΔΔCT^ method and normalized to the expression of the housekeeping gene GAPDH. Data presented are from two independent experiments in which all sample sets were analyzed in triplicate.

## Results

### Altered Thyroid Function in the TPOCreER2/iDTR Model

**Figure 2:**
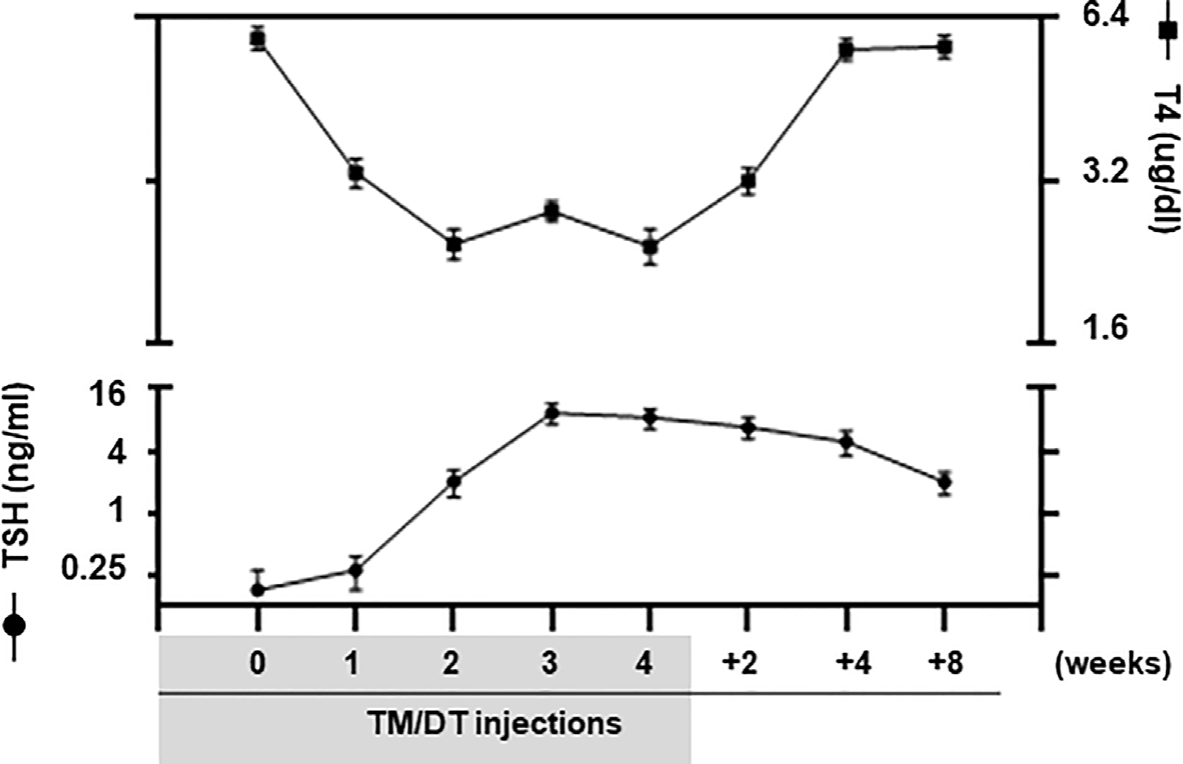
Thyroid function of TPOCreER2/iDTR mice. Blood samples were collected from pre-treatment, TM/TD treated mice at 1, 2, 3, 4 weeks; and at after stopping TM/TD treatment at 2, 4, 8 weeks later, as indicated on the X-axis. Serum total T4 (Right Y-axis) and TSH (Left Y-axis) were detected using multiplex bead assays (Millipore) as described in *Materials and Methods*. In the treated mice we found that by 2 weeks their serum T_4_ levels were markedly decreased after TM/DT administration and, at the same time, TSH levels had increased compatible with a significant degree of thyroid failure. Furthermore, after finishing 4 weeks of treatment, the thyroid function of the hypothyroid mice soon markedly improved. Serum T_4_ levels were restored to basal levels and in parallel TSH levels decreased although not yet back to normal at 8 weeks. Date are expressed as mean ± SD (n = 4–6). For comparison, TSHR KO mice (T4 0.30 ± 0.01 ug/dl; TSH 530.0 ± 45.0 ng/ml) and WT normal mice (T4 6.01 ± 0.06 ug/dl; TSH 0.25 ± 0.10 ng/ml) were used but not shown in the figure.

We used TM/DT treatment on TPOCreER2/iDTR mice as indicated in the scheme shown in **Figure 1** to damage the TPO-expressing mature thyroid follicular cells following intraperitoneal injection of 1mg TM and 100 ng DT/gram body weight on alternate days. The mice were observed daily over a 4-week period of TM/DT injections and their thyroid function monitored on weeks 2, 3, and 4. In the treated mice we found that by 2 weeks their serum T_4_ levels were markedly decreased (5.86 ug/dl in the controls to 2.43 ug/dl in the treated mice) after TM/DT administration and, at the same time, TSH levels had increased (from 0.25 to 8.39 ng/ml) compatible with a significant degree of thyroid failure (**Figure 2**). Furthermore, after finishing 4 weeks of treatment the thyroid function of the hypothyroid mice soon improved and returned to normal by another 8 weeks. Serum T_4_ levels were restored to basal levels (from 2.43 to 5.23 ug/dl) and in parallel TSH levels decreased (from 8.39 to 1.96 ng/ml) (**Figure 2**).

### Robust Depletion of Thyroid Cells in the TPOCreER2/iDTR Model After TM/DT Treatment

**Figure 3:**
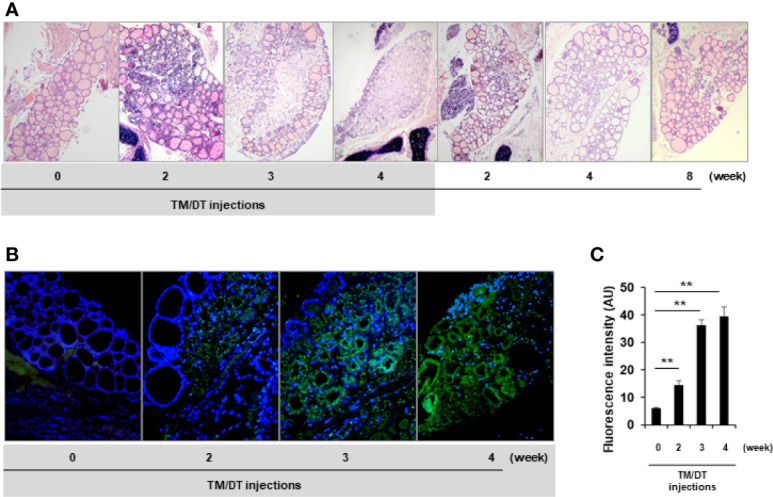
Histologic analysis of thyroid glands from TPOCreER2/iDTR mice. **(A)** H&E staining: Representative images of H&E analysis from control and TPOCreER2/iDTR mice following TM/DT administration and after stopping administration at different time points as indicated. Pre-injection (0 weeks): An intact thyroid gland is shown with well organized follicles; TM/TD injection (2, 3, and 4 weeks): Increasing damage to the gland is seen with a dramatic decrease in follicles observed with the normal structure of the thyroid gland almost extinguished; After stopping TM/TD injections (+2, +4, +8 weeks): The thyroid basic unit, the follicles, were observed to be increasing in number after stopping the TM/TD injections. **(B)** Apoptosis Analysis: Representative images of apoptosis staining from the control and TPOCreER2/iDTR mice following TM/DT administration at different time points as indicated. Pre-injection (0): No TUNEL positive cells in the thyroid follicles. TM/TD injection (2, 3, and 4 weeks): Abundant apoptotic TUNEL positive cells (green) observed with time. **(C)** Quantitative analysis of apoptosis in the thyroid: Apoptosis staining of TM/TD injected mouse thyroid as shown in B was quantified by image analysis software and expressed in arbitrary units (AU). At least three positive images were counted in each group and mean and SD were plotted. The positive staining after TM/TD injections at 2, 3, and 4 weeks were significantly higher than pre-injection (0) thyroids (p < 0.01)**.

Randomly selected mice were sacrificed after 2, 3, and 4 weeks of TM/DT treatment and their thyroid glands examined. We found that beginning at 2 weeks there was a marked inflammatory infiltrate and the majority of thyroid follicles became damaged in the TPOCreER2/iDTR mice but not in the vehicle treated control mice. Representative histology showed gross destructive changes with more than 90% of follicles damaged by 4 weeks. Thyroid follicles were still observed at the edges of the thyroid glands while the middle portion of the gland was totally ablated (**Figure 3A**). Furthermore, the same tissues showed substantial apoptotic cell death detected by TUNEL staining (**Figure 3B**). Quantitative assessment of the degree of apoptosis is shown in **Figure 3C**. After suspending the TM/DT administration, the histology of additional mice showed that the thyroid gland recovered with the formation of new thyroid follicles as early as 2 weeks and appeared as normal glands 8 weeks post treatment (**Figure 3A**).

### Decreased Thyroid Gene Expression

**Figure 4:**
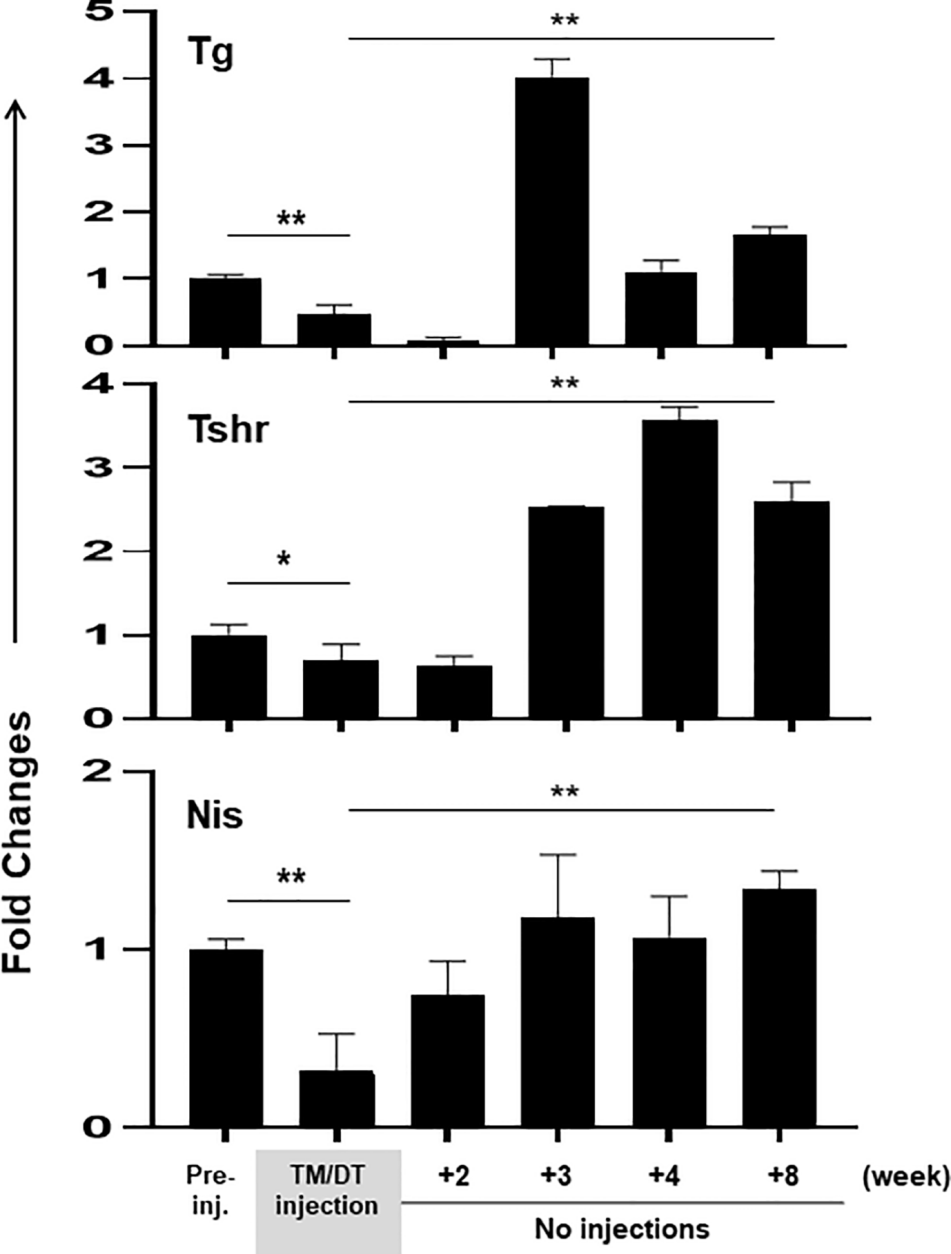
Thyroid gene expression in thyroid glands from TPOCreER2/iDTR mice: Total mRNA were isolated from the thyroid glands of each group of mice (n = 4–5) at time points as indicated on the X-axis. Five micrograms total RNA were used for the RT-PCRs. The relative expression of each transcript is presented as fold change compared to control non-injected samples (mean ± SEM of three independent experiments). The thyroid specific genes (TG, TSHR, and NIS) decreased after 4 weeks TM/TD treatment with an average ~50% fall in activity in comparison to controls (p < 0.05, or p < 0.01). After stopping treatment the gland showed a robust return to full thyroid gene activation, beginning after 2–3 weeks (p < 0.01)**.

At the same time as thyroid function was falling, the transcription of the thyroid specific genes (TG, TSHR, and NIS), when assessed by PCR, also decreased after 4 weeks with an average ~50% fall in activity **(** **Figure 4** **)**. These data also showed that at the time of the analysis, the thyroid gland structure was not totally destroyed, presumably there still remained non-apoptotic cells, which contributed to the remaining 50% gene transcription. However, after suspension of treatment the gland showed a robust, return to full thyroid gene activation, beginning after 2–3 weeks, and which appeared to be more active than before treatment and remained increased until the end of the study.

### A Stem Cell Surge During Mouse Thyroid Regeneration

**Figure 5:**
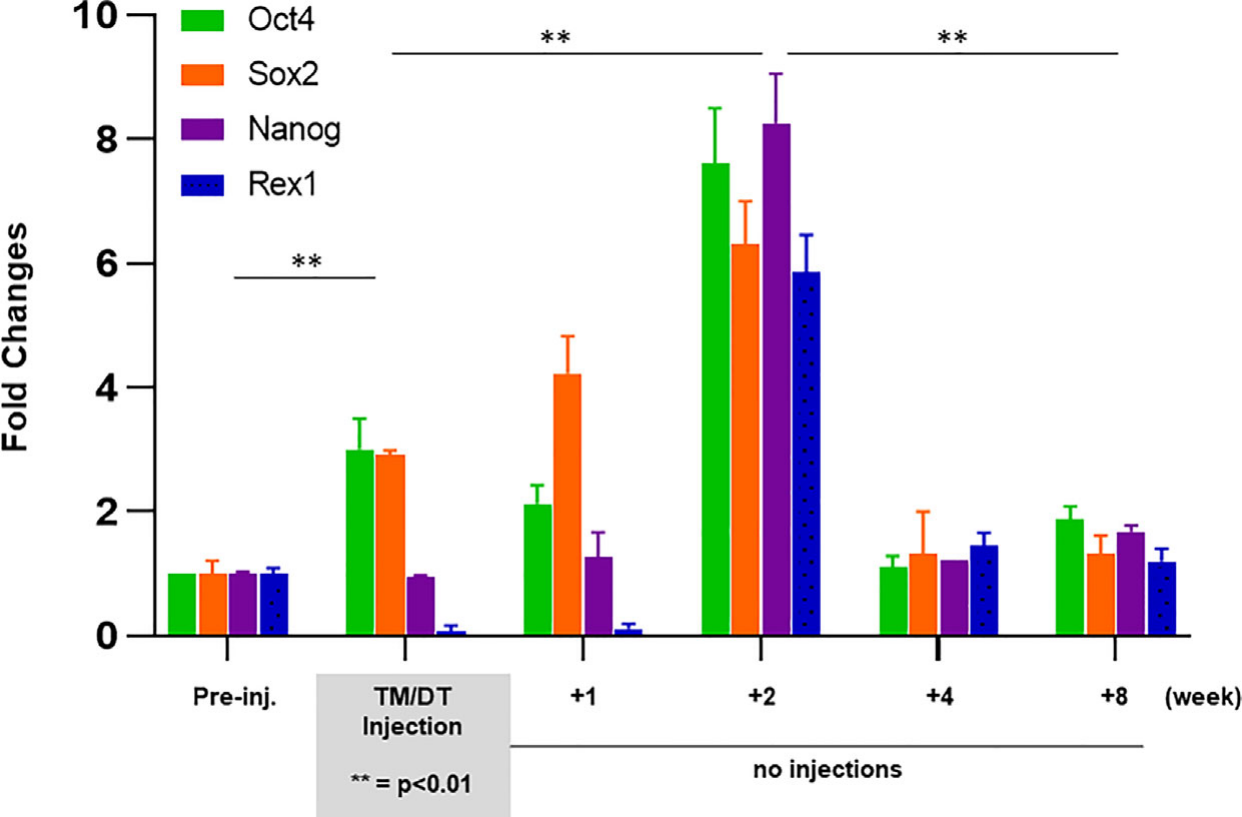
Expression of stem cell markers in thyroid glands from TPOCreER2/iDTR mice: Total mRNA were isolated from each group of mice (n = 4–5) as indicated on the X-axis. Five micrograms of total RNA were applied to RT-PCR. Relative expression of each transcript is presented as fold change compared to controls (mean ± SEM of three independent experiments). The stem cell markers (Ot4, Sox2) increased after 4 weeks TM/TD treatment (p < 0.01)**. After stopping treatment, all four of the stem cell markers (Ot4, Sox2, Nanog, Rex1) showed a robust increase at 2 weeks which then returned to control levels at 4 to 8 weeks post treatment.

In order to better understand the possible kinetic changes in resident stem cells in a thyroid gland recovering from TM/DT administration we assessed a series of stem cell markers in theTPOCreER2/iDTR mouse thyroids. There was a significant increase in stem cell markers during and following TM/DT treatment in comparison to the control mice **(** **Figure 5** **)**. Quantitative PCR at 1, 2, 4, and 8 weeks after stopping TM/DT administration revealed a transient surge in all stem cell markers at 2 weeks (Oct4, 7.6-fold increase; Nanog, 6.3-fold; Sox2, 8.2-fold; and Rex1, 5.8-fold), and then a return to the levels found in the earlier treated mice **(** **Figure 5** **)**.

**Figure 6:**
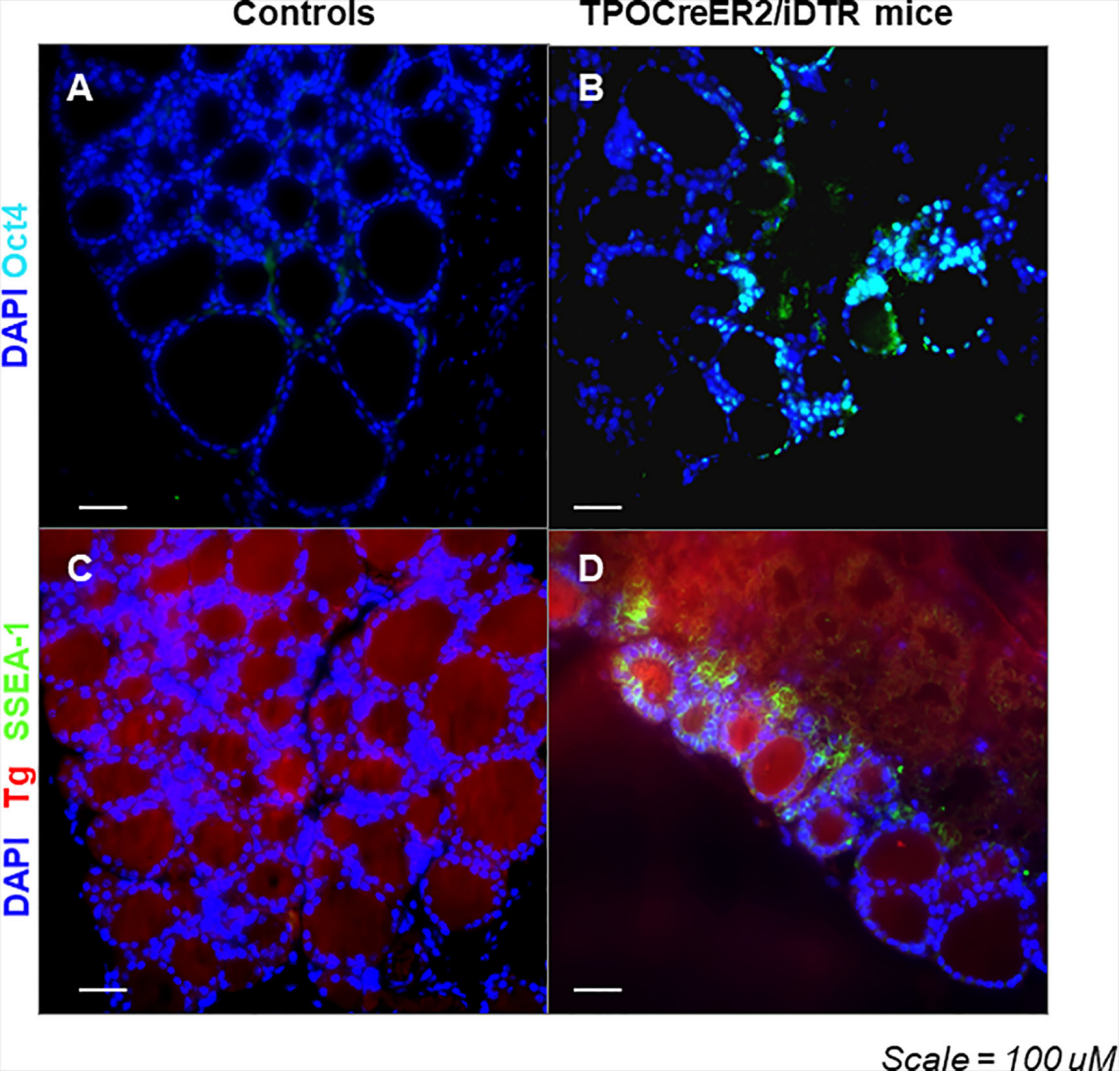
Expression of Oct4 and Ssea-1 in the thyroid glands from TPOCreER2/iDTR mice: **(A, B)** Representative images of positive immunostaining for expression of Oct4 (light blue) in TPOCreER2/iDTR mice injected with TM/DT for 2 weeks **(B)** compared to a negative control **(A)**. **(C, D)** Representative image of immunostaining for expression of Ssea-1 (green) and Tg (red) in TPOCreER2/iDTR mice injected with TM/DT for 2 weeks **(D)** compared with control thyroid **(C)**.

**Figure 7:**
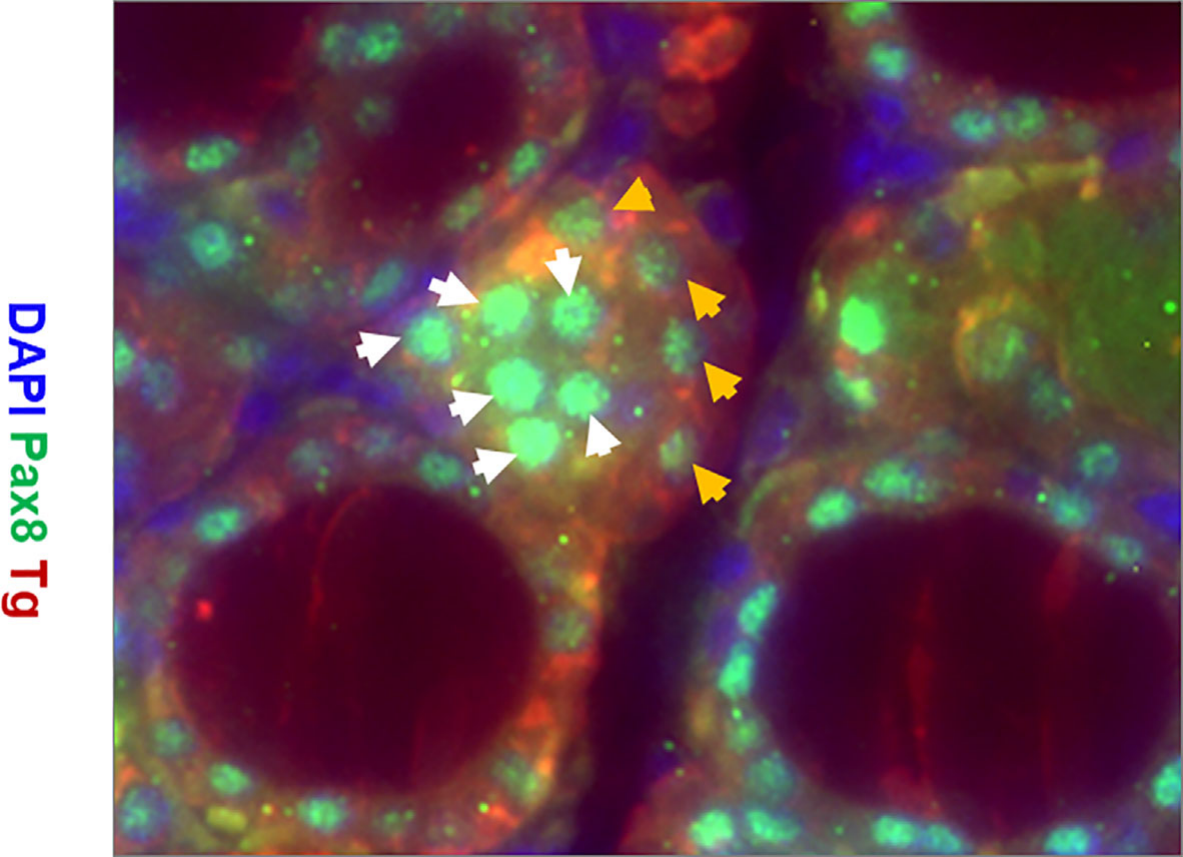
Representative images of immunostaining for expression of Tg (red), Pax8 (green), and DAPI (blue) in the thyroid gland of TPOCreER2/iDTR mice injected with TM/DT for 2 weeks. Note the larger parafollicular collections of cells positive for Pax8 (green) and negative for Tg (labeled with white arrows) indicative of progenitor cells and Pax 8 positive and Tg positive cells (labelled with orange arrows) indicative of more mature cells. There are also Pax8 and Tg negative cells (blue) which are likely fibroblast or other cell types.

Immunostaining analysis showed there were cells expressing stem cell markers such as Oct4 (**Figure 6B**) and Ssea-1 (**Figure 6D**) at 4 weeks post treatment in the TPOCreER2/iDTR mouse thyroids, often in clusters near maturing thyroid follicles as indicated but not in the controls (**Figures 6A, C**). Furthermore, we identified resident thyroid progenitor cells by their expression of Pax8 without thyroglobulin expression (**Figure 7**).

## Discussion

Most organs or tissues utilize resident stem cells that are critical for normal replacement and repair of damaged tissue. These cells have been well characterized in several tissues such as bone marrow, skeletal muscle, adipose tissue, skin, and the central nervous system (21–25). Resident stem cells are typically affiliated with a niche environment and remain in a quiescent state in adult tissues until reactivated for replacement or repair of damage (26, 27). In this study, we have traced the existence and dynamics of the resident thyroid stem cells using a novel thyroid injury model with transgenic TPOCreER2/iDTR mice. As described in the study, we developed the TPOCreER2/iDTR mice by crossing TPOCreER2 mice to ROSA26iDTR mice and used it to induce hypothyroidism. The advantage of this approach, with TPOCre-inducible expression of DTR, was that it renders only mature thyroid cells susceptible to ablation following Diphtheria Toxin (DT) administration. Thus, the TPOCreER2/iDTR mouse proved to be a useful model of controlled and inducible-targeted deletion of only mature thyroid follicular cells.

Studies from our laboratory (11, 28–30) and others (10, 31) have defined the potential of both embryonic and induced pluripotent stem cells to differentiate into thyroid follicular cells *in vitro*. However, finding the resident thyroid stem cells *in vivo* has been a challenge. Some earlier studies have tried to look at the cluster of side-population cells as a source of these stem cells in mouse thyroid (13) but these purified side population cells failed to show any major regenerative capacity. Some recent attempts have focused on finding the elusive thyroid “cancer stem cells” within the thyroid or after partial resection of the thyroid gland (5–7). In this regard the model we have used to study resident stem cells has the advantage of being an induced model where the nascent resident stem cells remain unharmed due to lack of TPO expression coupled with the advantage of selective ablation of mature thyroid cells. In addition, the contribution of extracellular matrix (ECM) signals that are much needed for the *in vivo* survival and proliferation of these reactivated stem cells is intact and recent studies have implicated the role of distinct non-stem cell factors (32) for regeneration in a context specific manner.

Using this model, parallel administration of TM and DT resulted in efficient ablation of most of the thyroid cells which was clearly indicated by a drop in all thyroid specific gene transcription (TG, TSHR, and NIS). Furthermore, we observed that the ablation of thyroid cells was due to apoptosis as observed by extensive TUNNEL staining following 4 weeks of toxin treatment which also led to the subsequent decrease in thyroid function with falling levels of serum T4 and increasing TSH by the end of 4 weeks. On further examination of the thyroid and its function it appeared that the hypothyroidism that was induced was not complete using the current protocol of TM/DT. The serum T4 decreased from ~5.86 to ~2.43 ug/dl and TSH increased from ~0.18 to ~8.39 ng/dl but the level of T4 was still higher and the level of TSH was still lower than that found in our TSHR knock-out mice where the T4 is as low as 0.03 ug/dl and TSH enormously high (20). Partial thyroidectomy models (4–6) have shown similar decreases in thyroid gene expression and decreased levels of thyroid hormone akin to this model suggesting monitoring of these parameters can be a reasonably good marker in judging the decrease in thyroid follicular load. Hence, there remained considerable thyroid functional capacity despite the high expression of the DT receptor in these mouse thyroids. Histological examination revealed an interesting pattern of cell destruction most severe in the central portion of the glands while peripheral follicles remained more intact. This may have been due to developmental or blood supply differences but also suggested that the DT receptor expression may have been more variable than we anticipated in these mice. The mechanism for the cell destruction after administration of TM/DT for 2-weeks was due to abundant thyroid cell apoptosis in the thyroid glands and after 4-weeks it was hard to identify normal thyroid follicle structures in many central areas of the thyroid glands. This severe destructive process was followed by a rapid recovery after suspending administration of TM/DT and histologically we observed thyroid follicle structures reforming and thyroid function was soon restored. By 8 weeks post treatment the thyroid function was well on its way to normality. This rapid functional recovery and thyroid gland regeneration appeared to be similar to that observed after partial thyroidectomy, experimental autoimmune thyroiditis (EAT), and in transgenic mice (3, 5–8). Furthermore, we know that embryonic development of the thyroid gland from the pharyngeal pouch is also completed rapidly by E16-18 in the mouse (33).

Temporal analysis of the thyroid glands following cessation of TM/DT administration at 1, 2, 4, and 8 weeks revealed a transient surge in stem cell markers at 2 weeks, and which rapidly returned to baseline. It is not uncommon that resident stem cells in tissues that are maintained in a quiescent state are awakened from their cell cycle arrested state to an active proliferative state on injury (27) but the molecular signatures that lead to the release of this quiescent state of resident stem cells in our model remains to be explored. In addition, immunohistochemical analysis showed there were para-follicular cell clusters stained with anti-Oct4 and anti-Ssea-1 in the recovering TPOCreER2/iDTR mice not seen in the controls and we could identify some apparent progenitor cells by their expression of Pax8 without thyroglobulin production in the recovering areas. This surge in stem cell activity was then followed by a rapid restoration of thyroid function indicating the role that thyroid stem cells play in regeneration of the damaged thyroid.

**Figure 8:**
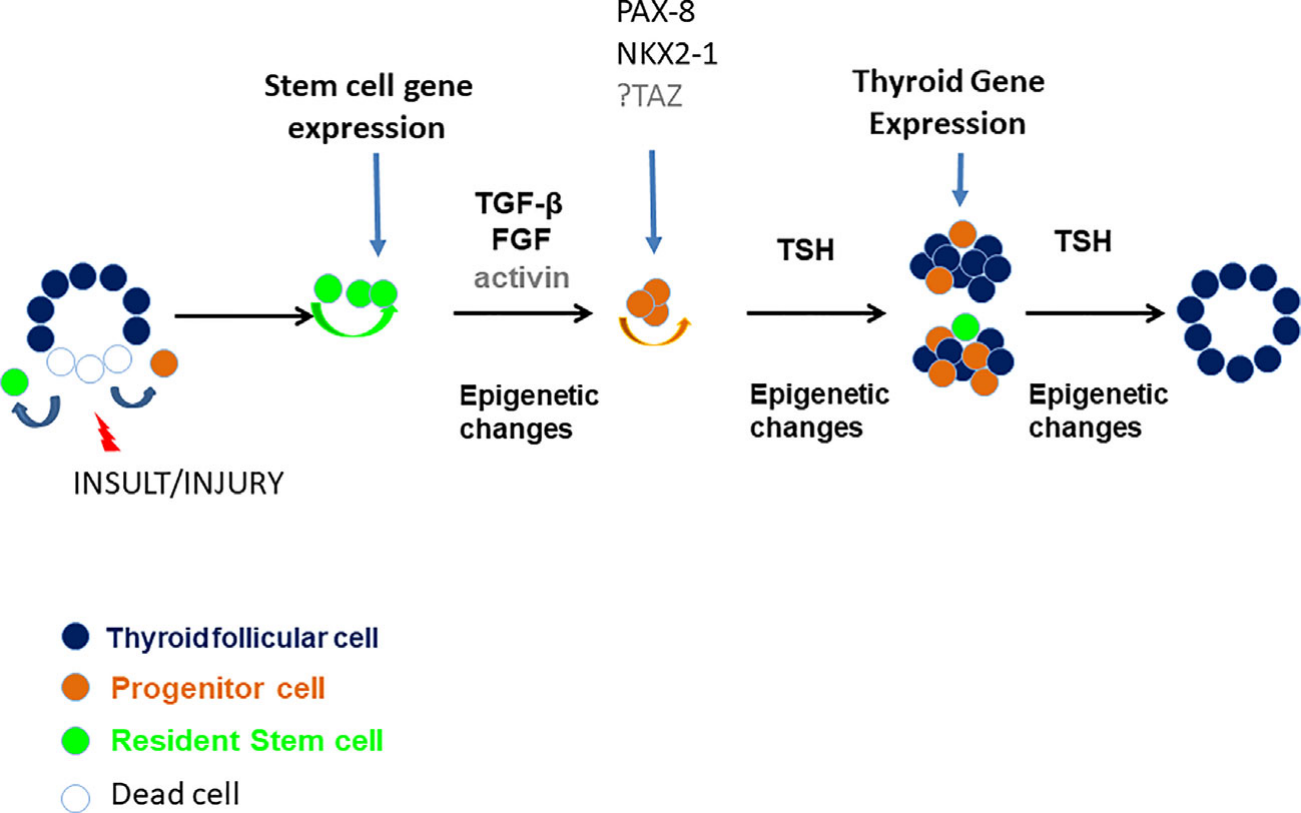
A proposed model of thyroid gland damage and regeneration. Various insults and injuries such as xenobiotics or pathophysiological conditions (i.e. iodide deficiency, autoimmune disease, partial thyroidectomy, etc.) may damage thyroid follicles and induce immature thyroid cells (such as resident stem cells or resident progenitor thyroid cells) to self-renew and/or differentiate through unclear mechanisms but likely involving TGF-β, FGF, activin, and TSH and involving epigenetic modifications resulting in repair/regeneration of a damaged thyroid gland.

Putting this information into the context of our current understanding generates a clear model of thyroid recovery after injury (**Figure 8**). The TM/DT model will allow the further exploration of each of the stages needed for successful thyroid gland repair. In conclusion, this study demonstrated a new model of initiating thyroid tissue regeneration in an organ that shows poor innate regeneration. This mouse model has helped us to identify a stem cell surge of thyroid resident stem cells by eliminating fully differentiated and mature thyroid follicular cells and has demonstrated the major role such cells play in thyroid regeneration.

## Data Availability Statement

The original contributions presented in the study are included in the article/**Supplementary Material**. Further inquiries can be directed to the corresponding author.

## Ethics Statement

The animal study was reviewed and approved by the Institutional Animal Care and Use Committees of the Icahn School of Medicine at Mount Sinai.

## Author Contributions

RM conceived the work, designed and performed experiments, and wrote the manuscript. SM designed and reviewed paper. RL conceived the work, designed experiments, and wrote the manuscript. TD conceived the work, designed experiments, and wrote the manuscript. All authors contributed to the article and approved the submitted version.

## Funding

Supported in part by grant DK069713 from the National Institutes of Health, a VA Merit Award (to TD), and the Segal Family fund.

## Conflict of Interest

The authors declare that the research was conducted in the absence of any commercial or financial relationships that could be construed as a potential conflict of interest.

## Supplementary Material

Supplementary Figure 1 Representative genotyping of TPOCreER2/iDTR mice by tail DNA. **(A)** The PCR for the DTR shows a 650 bp band for wild type and 340 bp band for mutant. **(B)** The PCR for Cre shows a 324 bp band for an internal control and 130 bp band for Cre.

Supplementary Figure 2 Analysis of DTR expression in the thyroid glands of TPOCreER2/iDTR mice. **(A)** qPCR analysis of the DTR in TPOCreER2/iDTR mouse thyroid and control thyroid. **(B)** The RT-PCR products in 2% agarose gel.

Supplementary Figure 3 Release of thyroglobulin from damaged thyroid follicular cells. This figure illustrates the increased serum thyroglobulin levels from TPOCreER2/iDTR. mice treated with TM/DT for 2 and 4 weeks when compared to control mice.
