# Supplementary figures and images for: A Stem Cell Surge During Thyroid Regeneration
Source: Front Endocrinol (Lausanne). 2021 Jan 21;11:606269. doi: 10.3389/fendo.2020.606269 (PMC7859487; doi:10.3389/fendo.2020.606269)

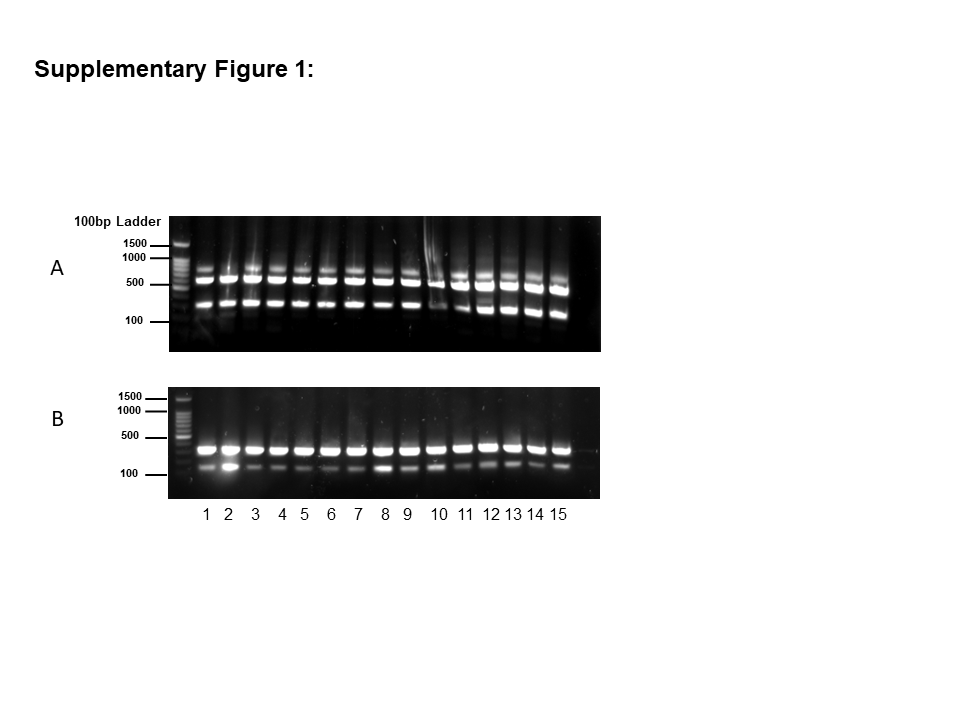

Supplement: Supplementary Figure 1 — Representative genotyping of TPOCreER2/iDTR mice by tail DNA. (A) The PCR for the DTR shows a 650 bp band for wild type and 340 bp band for mutant. (B) The PCR for Cre shows a 324 bp band for an internal control and 130 bp band for Cre. [file Image_1.tif]

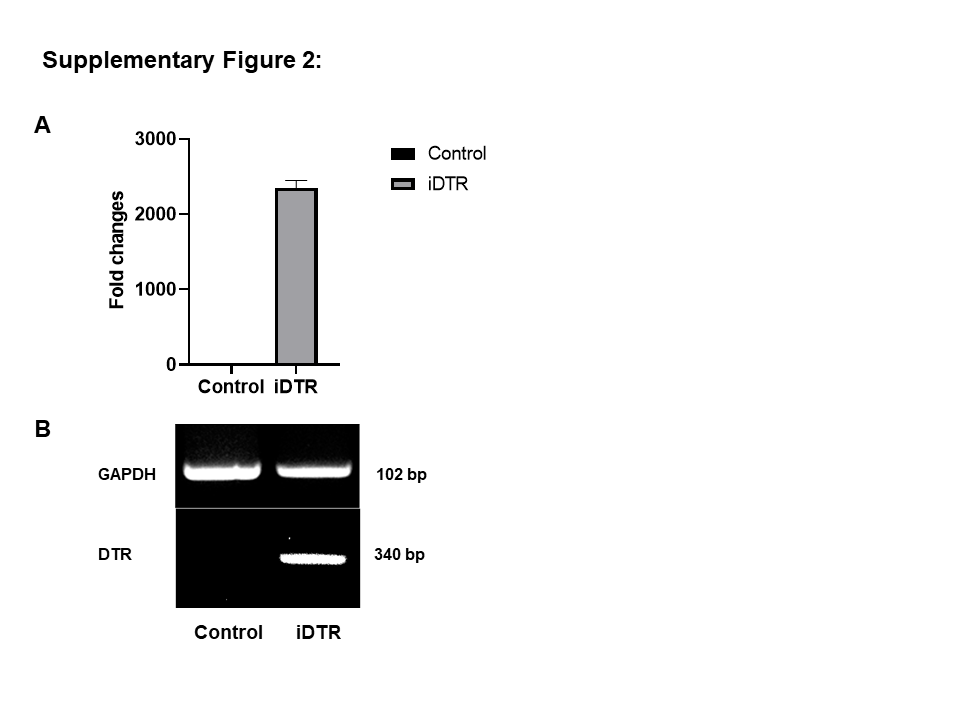

Supplement: Supplementary Figure 2 — Analysis of DTR expression in the thyroid glands of TPOCreER2/iDTR mice. (A) qPCR analysis of the DTR in TPOCreER2/iDTR mouse thyroid and control thyroid. (B) The RT-PCR products in 2% agarose gel. [file Image_2.tif]

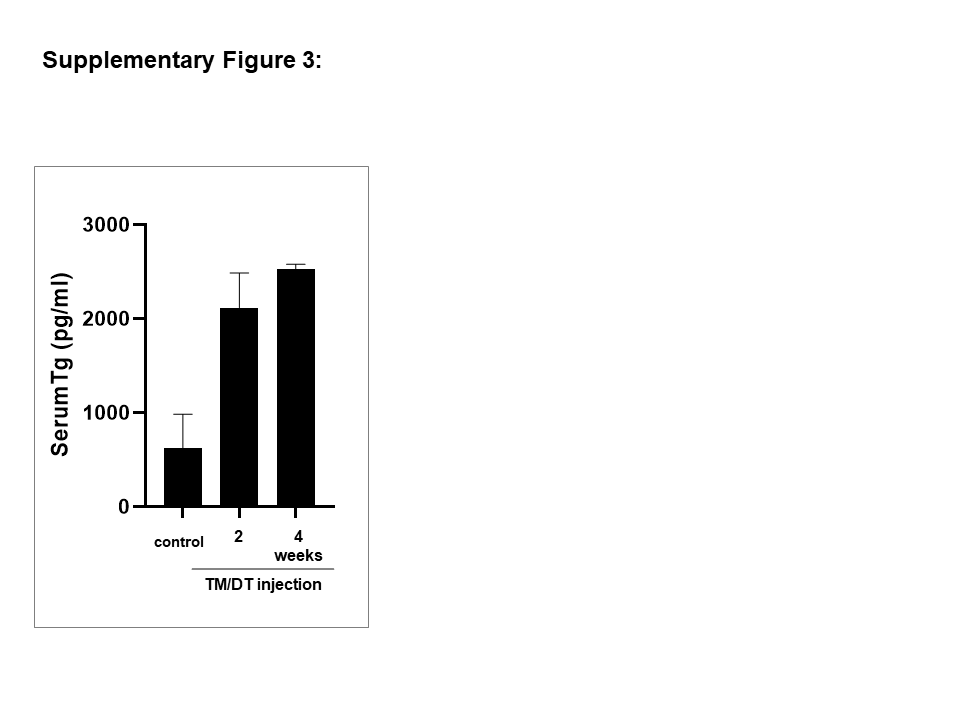

Supplement: Supplementary Figure 3 — Release of thyroglobulin from damaged thyroid follicular cells. This figure illustrates the increased serum thyroglobulin levels from TPOCreER2/iDTR. mice treated with TM/DT for 2 and 4 weeks when compared to control mice. [file Image_3.tif]
