# Supplementary material for: A Stem Cell Surge During Thyroid Regeneration
Source: Front Endocrinol (Lausanne). 2021 Jan 21;11:606269. doi: 10.3389/fendo.2020.606269 (PMC7859487; doi:10.3389/fendo.2020.606269)
Supplement: Supplementary file 4 [file Table_1.docx]

**Supplement Table 1. List of primers**

| **Gene Symbol**  **(Ref Seq #)** | **Amplicon Length** |  | **Start** | **Sequence** |
| --- | --- | --- | --- | --- |
| GAPDH (NM_008084) | 97 | Forward | 64 | CCCTTGAGCTAGGACTGGATAA |
|  |  | Reverse | 139 | GGGCTGCAGTCCGTATTTATAG |
| Tg  (NM_009375) | 102 | Forward | 1260 | AGGAACTCTTTGTTGACTCTGG |
|  |  | Reverse | 1340 | CTGCTCTGATGGCTTCTCTTAG |
| Tshr  (NM_011648) | 109 | Forward | 1407 | CTGCTAACCAGCCACTACAA |
|  |  | Reverse | 1494 | GGTCTACAGAGGCAATGAGAAG |
| Nis  (NM_053248) | 103 | Forward | 751 | GTGCTCAGTCTCGCTCAAA |
|  |  | Reverse | 833 | GGGAGCCACCTACTACAAATG |
| Tpo  (NM_009417) | 126 | Forward | 409 | GTTCTCCACGGATGCCTTATC |
|  |  | Reverse | 514 | GTGATGGGCCGGTACTTATTT |
| Nkx2-1  (NM_009385) | 84 | Forward | 176 | CGCCCAATTGAAGCAGAATG |
|  |  | Reverse | 240 | TCTAAAGTGGCGGCGTAAAT |
| Pax8  (NM_011040) | 90 | Forward | 722 | CCTTGGGCTCTACCTACTCTAT |
|  |  | Reverse | 789 | CCTGGTCACTATCATCCATCTTT |
| Foxe1  (NM_183298) | 100 | Forward | 1675 | AGGCACGAAACCTCACTAAC |
|  |  | Reverse | 1754 | GCACGTGAGTCTCAGTACAAT |
| Oct4  (NM_001252452) | 102 | Forward | 414 | GAGTCTGGAGACCATGTTTCTG |
|  |  | Reverse | 493 | CATACTCGAACCACATCCTTCTC |
| Nanog  (NM_028016) | 122 | Forward | 408 | GGCAGCCCTGATTCTTCTAC |
|  |  | Reverse | 510 | GAGAACACAGTCCGCATCTT |
| Sox2  (NM_011443) | 122 | Forward | 124 | GGAGAGAGAAAGAAAGGAGAGAAG |
|  |  | Reverse | 225 | CGAAGTGCAATTGGGATGAAA |
| Rex1  (NM_009556) | 108 | Forward | 83 | GGTACGAGTGGCAGTTTCTT |
|  |  | Reverse | 169 | CCCAGCTCTTAGTCCATTTCTC |
